# Anatomy and Biomechanics of the Deltoid Ligament Complex in Healthy Ankles: Protocol for a Systematic Review and Meta‐Analysis

**DOI:** 10.1002/jfa2.70198

**Published:** 2026-08-15

**Authors:** Linh Nguyen, Adam L. Bryant, Scott C. Starkey, Patrick L. Rowe, Allan Kent, Bryce A. Killen, Quentin A. Fogg, Kade L. Paterson

**Affiliations:** ^1^ Department of Physiotherapy Centre for Health, Exercise and Sports Medicine Melbourne School of Health Sciences The University of Melbourne Melbourne Victoria Australia; ^2^ College of Sport, Health and Engineering Victoria University Melbourne Australia; ^3^ Department of Movement Sciences KU Leuven Leuven Belgium; ^4^ Melbourne Victoria Australia

**Keywords:** anatomy, ankle injuries, biomechanics, chronic ankle instability, clinical management, computational modelling, Deltoid ligament complex

## Abstract

**Introduction:**

Up to one in two individuals who have a history of an ankle injury will develop chronic ankle instability, which subsequently increases the risk of osteoarthritis development. Although lateral ankle ligament injuries are the most common, recent research shows that concomitant injuries to the deltoid (medial collateral) ligament complex may be more prevalent than previously recognised. However, the anatomy and biomechanics of the deltoid ligament complex are reported inconsistently in the literature. This systematic review will summarise current evidence on the anatomy and biomechanics of the deltoid ligament complex in healthy adult ankles.

**Methods:**

Searches will be conducted in Scopus, MEDLINE, Embase, CINAHL and SPORTDiscus. Our search strategy will cover terms associated with ‘deltoid ligament’, ‘anatomy’ and ‘biomechanics’. We will only include dissection and imaging studies published in English that report any of the listed clinically relevant properties of the deltoid ligament in healthy adult human ankles. Two reviewers will independently perform screening and assess study quality using the anatomical quality assessment (AQUA) tool. One reviewer will extract relevant data, which will be independently verified by co‐authors. Primary outcomes include band prevalence, length, width, cross‐sectional area, maximum and/or failure load and elastic modulus. If three or more studies report a primary outcome, we will conduct a meta‐analysis and report findings as pooled means with 95% confidence intervals. If a meta‐analysis is not feasible, outcomes will be summarised as a narrative analysis. Measures will be taken during data synthesis to address anticipated methodological heterogeneity across included studies, and pooled estimates will be interpreted with caution.

**Discussion:**

This protocol details a systematic review that aims to summarise the anatomy and biomechanics of the deltoid ligament complex. Our findings will inform computational modelling, clinical management and biomechanics for ankle pathologies, as well as identifying research gaps and directions for future research.

**Trial Registration:**

PROSPERO: CRD420251142867

## 1 Introduction

The ankle is a common site of musculoskeletal injury, particularly among active populations [1]. Between a quarter to a half of individuals who sustain an ankle injury experience ongoing symptoms such as pain, limited range of motion and chronic ankle instability (CAI) [2, 3]. This may increase the risk of recurrent injuries and lead to early onset of osteoarthritis [1, 2, 3]. Individuals who participate in high‐intensity multidirectional sports (e.g., soccer, basketball, netball and volleyball) are shown to be at the highest risk of suffering repeated ankle injuries (50%–61% prevalence) [4]. Although most ankle injuries affect the lateral ligaments, medial structures like the deltoid (medial collateral) ligament complex are often overlooked. This is despite recent research indicating their importance in lateral and rotational stability [5, 6, 7, 8]. Retrospective studies also show that 44%–72% of patients who underwent surgery for CAI had undiagnosed deltoid ligament injuries on imaging [5, 9]. The underdiagnosis of deltoid injuries may be attributed to the limited range, low sensitivity and specificity of clinical tests [10, 11], which is due to the complex and poorly understood anatomy and mechanical role of the deltoid ligament complex [12, 13]. Untreated or mismanaged deltoid injuries can lead to serious long‐term sequelae, such as CAI and osteoarthritis [14], emphasising the need for a greater understanding of its anatomy, function and pathomechanics.

The deltoid ligament complex primarily resists subtalar joint eversion and external rotation [13], and hence may be injured when forces from these movements exceed tissue capacity. The ligament complex is typically reported to consist of a superficial layer comprising the tibionavicular, tibiocalcaneal, tibiospring and superficial posterior tibiotalar ligaments; as well as a deep layer comprising the deep anterior tibiotalar and deep posterior tibiotalar ligaments [15, 16, 17]. However, dissection [18, 19, 20, 21] and imaging [22, 23, 24] studies have reported inconsistent and often conflicting findings regarding the number of bands comprising the ligament complex, their prevalence (i.e., how often each band appears in the population) and attachment sites. This could be attributed to the varying quality of dissection and imaging techniques, as well as differing anatomical terminology and interpretation. Furthermore, these variations have been largely overlooked by biomechanical studies, which often assume a fixed number of deltoid bands based on a small selection of studies [25, 26]. This underscores a dissonance between the anatomical variations and biomechanical functions of the deltoid ligament complex reported in the literature, which may contribute to the ambiguity in clinical practice and research regarding ankle injuries.

Researchers are increasingly using musculoskeletal and finite element computational models to evaluate the biomechanics of human movement [27]. These computational models can integrate medical imaging alongside kinematic, kinetic and electromyography data to estimate joint contact forces and soft tissue stresses and strains [28, 29, 30]. However, the development of foot and ankle models has historically been slower compared to other joints (e.g., knee, hip), likely due to the complex anatomy of the region [31]. Current models either cover a limited range of soft tissues [32], derive their anatomy from a single participant and/or rely on a single randomly selected study [29, 30, 33] or arbitrary values [34, 35, 36] to model soft tissue parameters (e.g., ligament strain‐force curves). These methods do not account for inter‐individual variations and increase selection bias, meaning model predictions are not as physiologically robust when compared to simpler joints such as the knee. Accurate anatomical and biomechanical data are needed to maximise the potential of foot and ankle models being used for clinical applications and research into the underlying mechanisms of ankle injuries.

To date, there has been no systematic review on the anatomy and biomechanics of the deltoid ligament complex. Although numerous narrative reviews [12, 13, 16, 37, 38, 39, 40], one scoping review [41] and one short meta‐analysis [42] have investigated the properties of the deltoid ligament complex, they have covered a limited selection of studies or properties at insufficient depth. This highlights the need for a better understanding of the anatomy and biomechanics of the deltoid ligament complex to inform research and clinical practice, optimise diagnostic testing and enhance conservative and surgical management for ankle pathology.

The main objective of this protocol is to describe a systematic review and meta‐analysis of the anatomical and biomechanical properties of individual deltoid components in healthy ankles.

## 2 Methods

The protocol for this systematic review was developed following the recommendations from the preferred reporting items for systematic review and meta‐analysis protocols (PRISMA‐P) 2015 statement [43] and the Cochrane Handbook for systematic reviews of interventions [44]. We also incorporated the evidence‐based anatomy guidelines for systematic reviews and meta‐analyses, where applicable [45]. Our protocol was registered with the international prospective register of systematic reviews (PROSPERO) on the 10th of September 2025 (CRD420251142867). The PRISMA‐P checklist is shown in Supporting Information S1. We will report any amendments to the protocol upon the publication of the review findings.

### 2.1 Eligibility Criteria

#### 2.1.1 Types of Studies

This review will focus on original, peer‐reviewed journal articles published in English. Reviews, book chapters, conference proceedings, editorials, surveys, notes, letters, errata, dissertations and retracted publications will be excluded. Given the mixed qualitative and quantitative nature of this review, the eligibility criteria were developed using the sample, phenomenon of interest, design, evaluation and research type (SPIDER) framework [46] as detailed below:

Sample: To investigate healthy adult human ankles, we will exclude any studies or subject cohort with a reported history, diagnosis or signs of foot and/or ankle pathology (e.g., ligament injury, bone fracture, flatfoot, arthritis, joint replacement or fusion). For in vitro studies, this criterion applies according to the specimen source: (i) surgically amputated specimens must specifically state that there is no ligament or joint injury to be included; and (ii) cadaveric specimens are excluded if they report any foot or ankle pathology (i.e., due to stricter donor privacy laws in some countries).

Phenomena of interest: We will only consider studies that report on individual bands of the deltoid ligament complex.

Design: We will examine dissection (in vitro) and imaging studies (in vivo and in vitro), both of which may employ biomechanical testing. We will exclude computational modelling studies (e.g., musculoskeletal, finite element models). These studies often collect anatomical data from only one or two subjects for model development or validation [7] (i.e., subject‐specific and not representative of the population) and/or report only a single parameter for one deltoid band with limited methodological details [47, 48, 49], making it difficult to assess rigour and compare methodology between studies. We will also exclude studies with less than three samples to minimise the effect of outliers.

Evaluation: Studies will qualify for inclusion if they report one or more of the following ligament parameters: (i) anatomical properties including band attachment sites, prevalence, length, width, thickness, footprint area, cross‐sectional area and distance from centre of insertions to relevant osseous landmarks; (ii) biomechanical properties including band maximum and/or failure load, maximum and/or failure strain, submaximal or physiological strain, elastic modulus (Young's modulus), mode of failure and ultimate tensile strength. To facilitate the meta‐analysis and comparison of deltoid bands, we will exclude studies that: (i) are purely qualitative (e.g., those that only describe attachment sites); (ii) lack focus on individual deltoid components (e.g., those that only address the entire deltoid ligament complex); (iii) fail to provide mean and standard deviation for measurements, or for which such data cannot be extrapolated; or (iv) do not state the sample size. For the third and fourth criteria, we will attempt to contact the authors for clarification before excluding any studies (see Missing data).

Research type: Mixed quantitative and qualitative methods.

Following recommendations from the Cochrane Handbook [44], we will proceed with caution when making *post‐hoc* decisions about excluding studies if unexpected issues arise. Any modifications to the eligibility criteria will be reported in the final review and may be accompanied by a sensitivity analysis.

### 2.2 Methods for Identification of Studies

We will conduct a comprehensive search of the literature across five electronic databases: Scopus, MEDLINE (Ovid), Embase (Ovid), Cumulative Index to Nursing and Allied Health Literature CINAHL (EBSCO) and SPORTDiscus (EBSCO). We will not search the Cochrane Central Register of Controlled Trials (CENTRAL) and other trial registers nominated by the Cochrane Handbook [44], given that this review does not involve an intervention or exposure.

**FIGURE 1:**
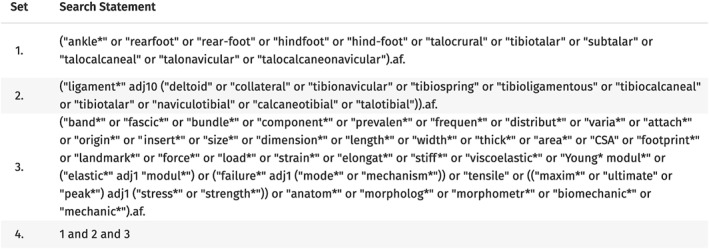
Search strategy for MEDLINE (Ovid).

We will employ the All Fields or All Text search option in all databases. The search terms were categorised according to three key criteria: (i) joints of interest, (ii) ligaments of interest and (iii) expected outcomes. The first criterion is particularly important as the term ‘collateral’ ligament is used interchangeably for ligaments found in the ankle, knee and elbow. We will utilise free‐text and indexed terms, proximity operators, common synonyms, truncations and variations of the sample, phenomena of interest and evaluation listed in the eligibility criteria above to ensure an optimal balance between search sensitivity and specificity. Other elements of the SPIDER framework (i.e., design and research type) were intentionally excluded from the search query to mitigate its potential limitation of having low search sensitivity [50]. The search terms were developed based on the preliminary search results, field knowledge, key studies [15, 21, 26] and relevant reviews [41, 42]. The search strategy for MEDLINE (Ovid) is shown in Figure 1, which was modified for other databases with the assistance of academic librarians. We will apply exclusion filters wherever available to remove publications that are not original journal articles published in English or animal studies, ensuring that we do not miss any publications that are yet to be indexed. No restrictions will be imposed on the publication date. The joint filters (e.g., knee, elbow) will not be used to minimise the likelihood of missing studies that involve multiple joints.

Additionally, we will examine the reference lists of included studies and relevant reviews to identify any eligible studies that do not appear in the database searches. We will not conduct any grey literature search as this academic field is relatively well‐established, and peer review is necessary to guarantee a minimum quality standard of the evidence considered for meta‐analysis [51].

### 2.3 Data Collection and Analysis

#### 2.3.1 Selection of Studies

One reviewer (LN) will perform database searches, retrieve the search results as RIS files and upload them to the Covidence systematic review software (Veritas Health Innovation, Australia), where duplicates will be automatically removed. Two reviewers (LN and AK) will independently conduct a two‐stage screening process: (i) titles and abstracts will be screened according to the pre‐defined eligibility criteria to identify potentially relevant studies; and (ii) full texts of the identified studies will be assessed for eligibility. Studies considered eligible by both reviewers will be included in the final review. Any conflicts between the two reviewers will be resolved through discussion. If a consensus cannot be reached, a third reviewer (KLP) will be consulted to make the final decision. Reviewers will not be blinded to the journal name, authors and publication year of the studies. However, reviewers will remain blinded to each other's screening decisions until a disagreement arises, upon which they will be notified by Covidence.

If necessary, we will contact authors for clarification regarding the study eligibility (see Missing data). We will record our search and screening results using a PRISMA flow diagram. During the data extraction phase, studies with the matching first author or multiple matching authors will be evaluated for potential data duplication. When there is reasonable suspicion that the same data is reported across different studies, reviewers will exclude any duplicate data with a lower methodological quality score (e.g., same demographic and/or baseline data). Authors will be contacted for clarification if any uncertainties arise regarding duplicate data.

#### 2.3.2 Data Extraction

One reviewer (LN) will extract all data, which will be independently verified by co‐authors. Any discrepancies will be resolved by discussion. We will utilise a structured, piloted data collection form in Microsoft Excel 365 (Microsoft Corp, USA) to extract the following data from each study (where available):

Study characteristics: First author, year of publication, title, journal, country where study was conducted and sample size (paired and/or unpaired ankles).

Participant characteristics: Ankle side (left and/or right ankles), age, sex, height, weight and foot length.

Study design: (i) specimen type (e.g., embalmed or fresh‐frozen) for in vitro studies; (ii) imaging modality (e.g., MRI) and planes for imaging studies; and/or (iii) range of motion tested, amount and/or direction of load applied, preconditioning and biomechanical model (e.g., whole specimen, bone‐ligament‐bone unit) for studies with biomechanical testing. The methods of measurement will also be collected for anatomical parameters (e.g., calliper, 3D digitiser) and biomechanical parameters (e.g., strain gauge).

Anatomical and biomechanical outcomes: We will extract the original names of reported deltoid bands and classify bands based on dissection or radiological images (if available), descriptions of their attachment sites, spatial relationships between bands and to nearby bony landmarks where necessary. Standardised anatomical nomenclature will be applied as follows: (i) each deltoid band will be named according to its proximal and distal attachments (e.g., tibiotalar ligament describes the fibres spanning between the tibia and talus); and (ii) where two or more bands share the same attachments, directional terms will be used to distinguish their sagittal positions (e.g., anterior and posterior tibiotalar ligaments) and depth (e.g., superficial and deep posterior tibiotalar ligaments) based on any reported spatial relationships and images [16, 52]. Following previous narrative and scoping reviews, we expect the deltoid ligament complex to be composed of a superficial layer (comprising the tibionavicular, tibiocalcaneal, tibiospring and superficial posterior tibiotalar ligaments) and a deep layer (comprising the deep anterior tibiotalar, deep intermediate tibiotalar and deep posterior tibiotalar ligaments) [16, 41, 42]. However, we will reconsolidate this band classification after all studies are extracted to ensure anatomical consistency. If any deltoid bands are reclassified, reannotated diagrams and original names of deltoid bands will be included in the appendix of the final review. Following the classification process, we will extract various parameters for each deltoid band: (i) primary outcomes will include (where available) parameters required for computational modelling, including band prevalence, length, width, cross‐sectional area, maximum and/or failure load and elastic modulus (Young's modulus); (ii) secondary outcomes may include other clinically relevant parameters such as band attachment sites, thickness, footprint area, distance from centre of insertions to relevant osseous landmarks (stratified by different landmarks used), maximum and/or failure strain, submaximal or physiological strain (stratified by ankle positions and range of motion tested), mode of failure (rupture or avulsion) and ultimate tensile strength. If available, measurements will be collected at both the midpoint and footprints of each band (e.g., width, thickness), or at both its midline and borders (e.g., length). For load and strain, maximal and failure values will be determined based on the authors' definitions and experimental protocols. If a direct value is not reported, we will calculate strain as the change in length (i.e., elongation) divided by the original length. If the elastic modulus is not reported, we will extract its derivative (e.g., stiffness). Mode of failure will be extracted as nominal data, such as rupture and avulsion. Attachment sites will be extracted as ordinal data, such as the medial, lateral, dorsal and plantar aspects of a particular bone.

We anticipate that all outcomes will be reported as continuous data, except for attachment sites and mode of failure. We will calculate the mean and standard deviation for each parameter in the standard units of measurement. If only the mean and 95% confidence interval are provided, the standard deviation will be calculated from these values to facilitate comparisons between studies.

### 2.4 Quality Assessment of Included Studies

We will evaluate the quality and potential bias of the included studies using the Anatomical Quality Assessment (AQUA) tool [53]. Five domains will be assessed: (i) objectives and subject characteristics, (ii) study design, (iii) methodology characterisation, (iv) descriptive anatomy and (v) reporting of results. For each study, two reviewers (LN and AK) will independently appraise each of the five domains as having low, high or unclear risk of bias. If any disagreement arises and the two reviewers cannot reach a consensus through discussion, a third reviewer (KLP) will be consulted to make the final decision. The risk of bias for all included studies will be summarised in a table and used to interpret the findings from the data analysis.

### 2.5 Missing Data

We will contact the authors if a study is missing important information required for data synthesis such as sample size, mean and standard deviation for measurements. If the authors do not respond or refuse the request for missing data, we will attempt to extract mean values and standard deviation from graphical figures using the Web Plot Digitizer software (Version 5.2, Rohatgi, 2024). If these efforts fail, we will consider excluding the corresponding studies or experiments. Authors will also be contacted to clarify any suspected case of duplicate data between different studies.

### 2.6 Data Synthesis and Analysis

We will pool data from included studies for each outcome of individual deltoid bands, irrespective of the investigation method (e.g., imaging, dissection). For primary outcomes, we will conduct a meta‐analysis if at least three studies are available for each data pool. This threshold was conservatively set above the minimum of two studies recommended by the Cochrane Handbook [44], given that anatomical and biomechanical studies often have small sample sizes. The meta‐analysis will be performed using the random‐effects model and inverse‐variance method to address the anticipated heterogeneity among studies [44, 54]. Outcomes will be reported as pooled means or prevalences with 95% confidence intervals, visualised in forest plots. We will consider the difference in properties between the two deltoid bands as statistically significant if the 95% confidence interval of one does not overlap with the pooled mean or prevalence of the other. *p*‐values may be calculated to determine the statistical significance between deltoid bands. Two reviewers (LN and AK) will independently assess the quality of the synthesised evidence using the Grading of Recommendations Assessment, Development and Evaluation (GRADE) approach as outlined in the Cochrane Handbook [44, 55]. A summary of findings table will be produced for each outcome using the GRADEpro software (McMaster University and Evidence Prime, Canada) to compare outcomes across different deltoid bands. Since all anatomical and biomechanical studies are observational rather than intervention‐based, we will assign all outcomes with an initial evidence grading of ‘medium quality’ and adjust the quality according to the factors outlined in the GRADE Handbook [55]. The GRADE framework will allow certainty of the synthesised evidence to be interpreted with caution, particularly given the expected high heterogeneity arising from the inclusion of a wide range of study designs (i.e., dissection and imaging studies), measurement techniques and specimen preparation.

We will assess the presence of heterogeneity using the chi‐squared test with a significance level of 10%. This will be followed by the I^2^ statistic to quantify the percentage of variability across studies attributed to heterogeneity rather than random chance. We will interpret I^2^ values of 30%, 50% and 75% as moderate, substantial and considerable heterogeneity, respectively [44, 55]. If any heterogeneity is identified, subgroup analyses and meta‐regression (see Subgroup analyses) may be performed to explore the potential sources of heterogeneity [44]. In the case of considerable heterogeneity, we may examine the possibility of reclassifying the deltoid bands, and pooled estimates will be interpreted with caution [44, 56]. To further investigate sources of variability, sensitivity analyses will be performed for: (i) studies with 20 or more samples; and (ii) studies that use surgically amputated specimens to determine whether the underlying cause of the amputation could affect pooled estimates. We chose not to set a maximum heterogeneity threshold for meta‐analysis as there is no consensus on an appropriate limit [56]. Instead, we will investigate the sources of heterogeneity through subgroup and sensitivity analyses, which may provide more insights on methodological and clinical comparability than avoiding meta‐analysis altogether.

Where meta‐analysis is not feasible, such as due to insufficient studies or secondary outcomes, we will perform a narrative synthesis. This approach involves examining the similarities and differences in the pooled results to identify potential patterns in the data. We will also compare the methodology, study characteristics and risk of bias level (i.e., AQUA score) across studies to explain potential variability in the results. These findings will be presented in a summary table [44, 57].

Statistical analyses will be performed using the Review Manager statistical software (RevMan, Version 7.2.0, The Cochrane Collaboration, 2024). Meta‐regression will be carried out in R statistical software (Version 4.5.2, R Core Team, Austria, 2025).

### 2.7 Assessment of Non‐Reporting Bias

For each data pool with at least 10 studies, we will construct a funnel plot to assess small‐study effects and identify any potential publication bias. Statistical tests will be performed to objectively evaluate the asymmetry of the funnel plots. Further sensitivity analyses may be required to investigate any asymmetry observed [44].

### 2.8 Subgroup Analyses

If there is a minimum of 10 studies available, we plan to perform subgroup analyses for imaging and dissection studies, geographical location of the study and level of methodological quality (i.e., AQUA score). This will be important for examining the sources of heterogeneity, given that the review covers both in vivo and in vitro studies with a diverse range of study designs.

We may also consider performing meta‐regression for subject age and sex, provided there are at least 10 studies to support this subgroup analysis [44].

## 3 Discussion

Current diagnosis and management of ankle injuries remain inadequate for many people, with up to one in two experiencing CAI and other debilitating sequelae [1, 2, 3]. This has recently been suggested to be due to the high rate of underdiagnosed concomitant deltoid ligament injuries [5, 9]. This underdiagnosis is at least partly due to the complex and poorly understood anatomy and biomechanical function of the deltoid ligament complex, which has resulted in a lack of accurate diagnostic tests. Therefore, a more comprehensive understanding of the deltoid ligament complex is necessary to guide clinical diagnosis, conservative and surgical management, and to inform research on the pathomechanics of ankle injuries. To our knowledge, this will be the first systematic review and meta‐analysis to examine the anatomical and biomechanical properties of a ligamentous structure in the human body.

The strength of our review will include its comprehensive coverage of a broad range of anatomical and biomechanical properties, focusing on individual deltoid components rather than the entire ligament complex. As such, we will be able to integrate anatomical findings from dissection and imaging studies with biomechanical behaviour, to provide a more holistic understanding of the ligament complex's role in ankle biomechanics and functions. We have developed our protocol based on the Cochrane and PRISMA guidelines [43, 44], and performed rigorous piloting for database searches and screening to ensure its robustness. However, we acknowledge there are limitations in the review process. First, this review will include only English‐language studies and will be restricted to adult subjects with healthy ankles. This approach was chosen as normal deltoid complex anatomy and biomechanics are not well understood. Second, a high level of heterogeneity is anticipated given that we are synthesising evidence from a range of study designs, specimen characteristics, measurement techniques and anatomical definitions. Thus, we have taken several precautions to address heterogeneity in statistical pooling: (i) excluding computational modelling studies due to a variety of study designs and insufficient methodological details; (ii) performing subgroup analyses (e.g., for dissection and imaging studies) and sensitivity analyses (e.g., for surgically amputated specimens) to determine whether variability in overall results are due to methodological heterogeneity; (iii) assessing certainty of synthesised evidence using the GRADE framework to account for heterogeneity; and (iv) reclassifying deltoid bands using standardised nomenclature to ensure anatomical consistency. Given that current computational models derive ligament data from a single study/subject [29, 30, 33] or arbitrary values [34, 35, 36] (i.e., high selection bias), we believe it may be more advantageous to investigate and acknowledge the sources of heterogeneity in our findings than to avoid meta‐analysis altogether [56].

Our systematic review findings will provide pooled estimates and a summary of the current evidence on the anatomy and biomechanics of the deltoid ligament complex. These insights will assist clinicians and researchers in optimising the diagnosis and treatment of ankle injuries, as well as identifying existing research gaps and directions for future studies.

## Author Contributions

**Linh Nguyen:** conceptualization, methodology, writing – original draft, writing – review and editing. **Adam L. Bryant:** conceptualization, methodology, supervision, writing – review and editing. **Scott C. Starkey:** conceptualization, methodology, supervision, writing – review and editing. **Patrick L. Rowe:** conceptualization, methodology, writing – review and editing. **Allan Kent:** methodology, writing – review and editing. **Bryce A. Killen:** writing – review and editing. **Quentin A. Fogg:** writing – review and editing. **Kade L. Paterson:** conceptualization, methodology, supervision, writing – review and editing.

## Funding

This research was supported by the Commonwealth through an Australian Government Research Training Program Scholarship [DOI: https://doi.org/10.82133/C42F‐K220].

## Conflicts of Interest

The authors declare no conflicts of interest.

## Supporting information


Supporting Information S1

## Data Availability

Data sharing is not applicable to this article as no datasets were generated or analysed during the current study.
